# Iridovirus Infection in Chinese Giant Salamanders, China, 2010

**DOI:** 10.3201/eid1712.101758

**Published:** 2011-12

**Authors:** Wuzi Dong, Xiaoming Zhang, Changming Yang, Junhui An, Jinzhou Qin, Fengfeng Song, Wenxian Zeng

**Affiliations:** Northwest A & F University, Yangling, People’s Republic of China (W. Dong, X. Zhang, J. An, J. Qin, F. Song, W. Zeng);; Tiancheng Giant Salamander Bioengineering Ltd, Hanzhong, People’s Republic of China (C. Yang)

**Keywords:** Chinese giant salamanders, Andrias davidianus, iridovirus, histopathology, neighbor-joining tree, viruses, People’s Republic of China

**To the Editor:** The Chinese giant salamander (*Andreas davidianus*) is one of the world’s largest amphibian species and is often referred to as a living fossil. They primarily inhabit drainage basins of the Yangtze River, the Yellow River, and the Pearl River in the People’s Republic of China (*1*). Because of habitat loss, pollution, and overharvesting, the population of wild Chinese giant salamanders has dropped sharply (*2**,**3*). As a result, the Chinese giant salamander is artificially farmed in mesocosms for research and conservation. The mesocosms (ambient temperature <20°C) are maintained primarily in mountainous caves and mountainous ditches. During June–October 2010, a high mortality rate was reported in salamanders in ditch mesocosms in Shaanxi, Sichuan, and Henan, reaching an epidemic peak in July. Mortality rate reached 95% in the affected areas. Although bacteria, including *Aeromonas hydrophila* (*4*), were isolated from sick salamanders, antimicrobial drug treatment did not successfully improve the situation. Further pathologic analysis and viral testing were subsequently performed.

Pathologic changes were similar among the affected salamander populations. Gross anatomical changes included palpebral hyperemia or swelling; mouth pouch erythema; ecchymoses in the oral cavity; petechiae, ulceration; and erythema on the dorsal and ventral body surface; toe necrosis (Technical Appendix Figure 1, panels A, B); emaciation; friable and gray-black liver; and mottled, friable lesions of the kidney and spleen (Technical Appendix Figure 1, panel C). Histologic examination showed hyperplastic lymphoid nodules in the spleen (Figure, panel A). Additionally, nuclear debris, macrophages (Figure, panel A), and intracytoplasmic inclusion bodies (Figure, panel B) were observed in the lymphoid nodules. Liver sinusoids were enlarged and contained large numbers of macrophages. Degenerating hepatocytes were noted (Technical Appendix Figure 1, panel D). Degenerate renal epithelial cells were shed from the basement membrane and were found in the lumen of the renal tubules (Technical Appendix Figure 1, panel E). A large number of viral particles were observed in renal epithelial cells (Technical Appendix Figure 1, panel G ). Virus was isolated from the liver, kidney, and spleen. Electron microscopy was performed on random tissue samples from organs positive for an unidentified virus. Icosahedral viral particles ≈150 nm in diameter were observed in the cytoplasm of some cells (Figure, panel B; Technical Appendix Figure 1, panels F, G ).

**Figure:**
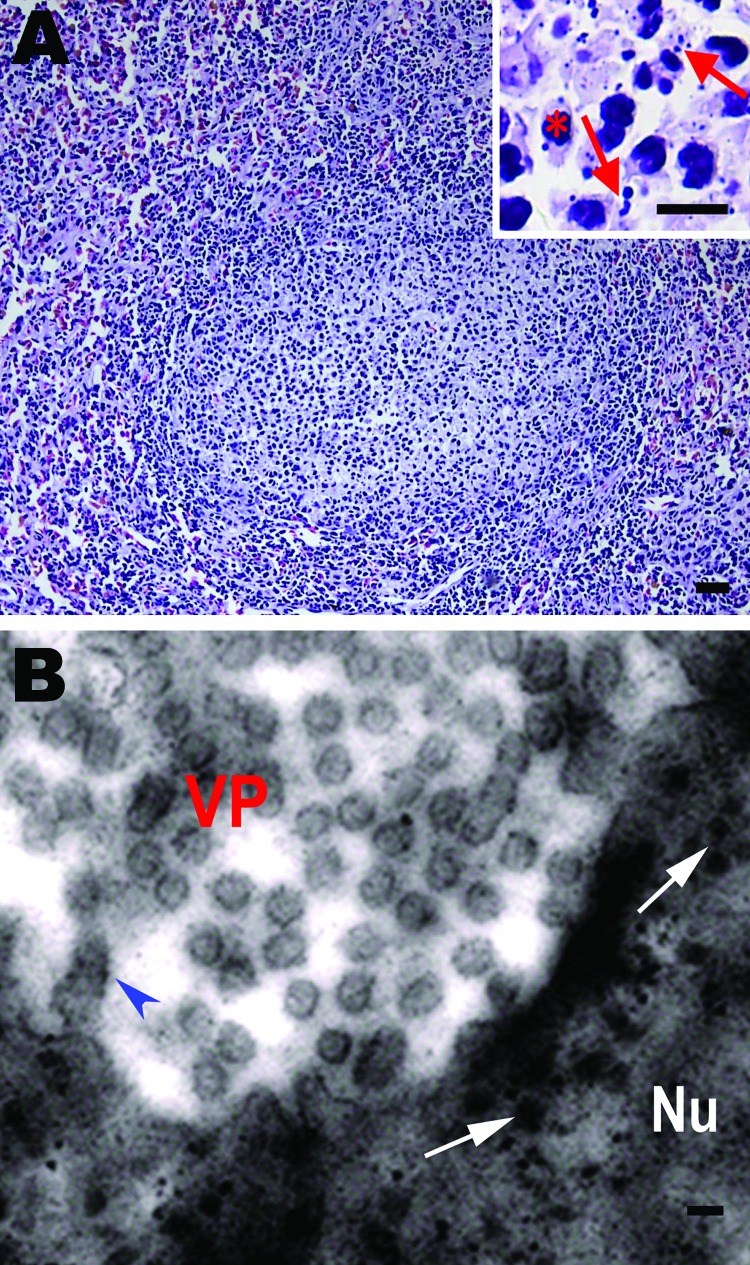
Histologic changes in the spleen of sick Chinese giant salamanders (*Andrias davidianus*), People’s Republic of China, 2010. Electron microscopy shows virus particles in splenocytes. A) Hyperplasia of lymphoid nodules in the splenic white pulp. Inset: Some splenocytes contain nuclear debris (arrows) and macrophages (asterisk). Hematoxylin and eosin stain; scale bars = 80 μm. B) Electron microscopy image of viral particles in splenocytes. Many viral particles are cytoplasmic and appear hexagonal or round. Scale bar = 200 nm. VP, viral particles in cytoplasm; Nu, nucleus; arrowhead, provirus in nuclear membrane; arrows, provirus in nucleus.

On the basis of the gross lesions and the appearance of the virus, we suspected that it was a member of the iridovirus family. To test this hypothesis, genomic DNA (gDNA) was extracted from the isolated virus by using a commercial kit (Genray, Shanhai, China). PCR was performed by using 3 sets of primers targeting 681 bp, 568 bp, and 616 bp iridoviral fragments respectively, from the major capsid protein gene (GenBank accession no. U36913; 5′-CCCCTCCCATTCTTCTTCTCC-3′, 5′-GGCGTTGGTCAGTCTACCGTAAT-3′), the ATPase gene (GenBank accession no. M80551; 5′-CCAAGAGGCACATCATACCG-3′, 5′-GCTGGACATCTCCTACGACCC-3′), and the thymidine kinase gene (GenBank accession no. AY837779; 5′-GGGCTAATGTATTGAAGACGC-3′, 5′-TTGTAAACTTGGAGTGGAGGG-3′). Resulting PCR products from 10 salamanders were sequenced and compared with the corresponding sequences of the 5 known iridovirus strains by using a BLAST search (http://blast.ncbi.nlm.nih.gov/Blast.cgi) (frog virus 3, GenBank accession no. AY548484; soft-shelled turtle iridovirus, GenBank accession no. EU627010; tiger frog virus, GenBank accession no. AF389451; epizootic hematopoietic necrosis virus GenBank accession no. FJ433873; and Ambystoma tigrinum stebbensi virus, GenBank accession no. AY150217). The sequences of the 3 PCR products from the virus-infected Chinese giant salamanders (GenBank accession nos. HQ829176, HQ829177, and HQ829178) showed >96% homology with the corresponding sequences of the 5 iridovirus strains. Additionally, neighbor-joining tree analysis showed that the virus was clustered in 1 lineage with frog virus 3, soft-shelled turle iridovirus, and tiger frog virus (Technical Appendix Figure 2 ). These results suggest that the high mortality rates in Chinese giant salamanders were caused by a virus in the iridovirus family.

The iridoviruses are carried in the bodies of vertebrates such as gopher tortoises (*Gopherus polyphemus*) (*5*), Chinese forest frogs (*Rana dybowskii*) (*6*), and fish (*7**,**8*). Iridoviruses are thought to be transmitted horizontally in lower vertebrates such as bullfrogs (*7**,**9**,**10*). In addition, some iridovirus infections may be chronic or conditional (*7*). In this study, the virus was isolated from the liver and spleen of 30 sick (n = 7) or dead (n = 23) salamanders that were farmed in ditch mesocosms, where ambient temperatures were unusually high (>25°C) at the time of the epidemic. Although the virus also was isolated from animals living in cooler cave mesocosms (ambient temperature <18°C), these animals showed no apparent signs of illness. Studies have reported that, when infection is detected early in the course of the disease and when exogenous stress is minimized, mildly affected bullfrogs are able to clear the virus (*9**,**10*). The high water temperatures in the ditch mesocosms (i.e., >25°C) and the associated stress on the animals may have increased disease in ditch-dwelling Chinese giant salamanders. This seems particularly likely, given the absence of clinical signs of disease in infected salamanders that lived in the cooler cave mesocosms (i.e., <18°C). In addition, absence of exposure of Chinese giant salamanders to other animal carriers of the virus may prevent horizontal transmission of iridovirus.

## Supplementary Material

Technical Appendix Gross anatomic and histologic changes in sick Chinese giant salamanders (*Andrias davidianus*), People's Republic of China, 2010.
