# Supplementary material for: Iridovirus Infection in Chinese Giant Salamanders, China, 2010
Source: Emerg Infect Dis. 2011 Dec;17(12):2388–9. doi: 10.3201/eid1712.101758 (PMC3311219; doi:10.3201/eid1712.101758)
Supplement: Technical Appendix — Gross anatomic and histologic changes in sick Chinese giant salamanders (Andrias davidianus), People's Republic of China, 2010. [file 10-1758-Techapp_2p.pdf]

Article DOI: http://dx.doi.org/10.3201/eid1712.101758

Iridovirus Infection in Chinese Giant Salamanders, China, 2010

Technical Appendix

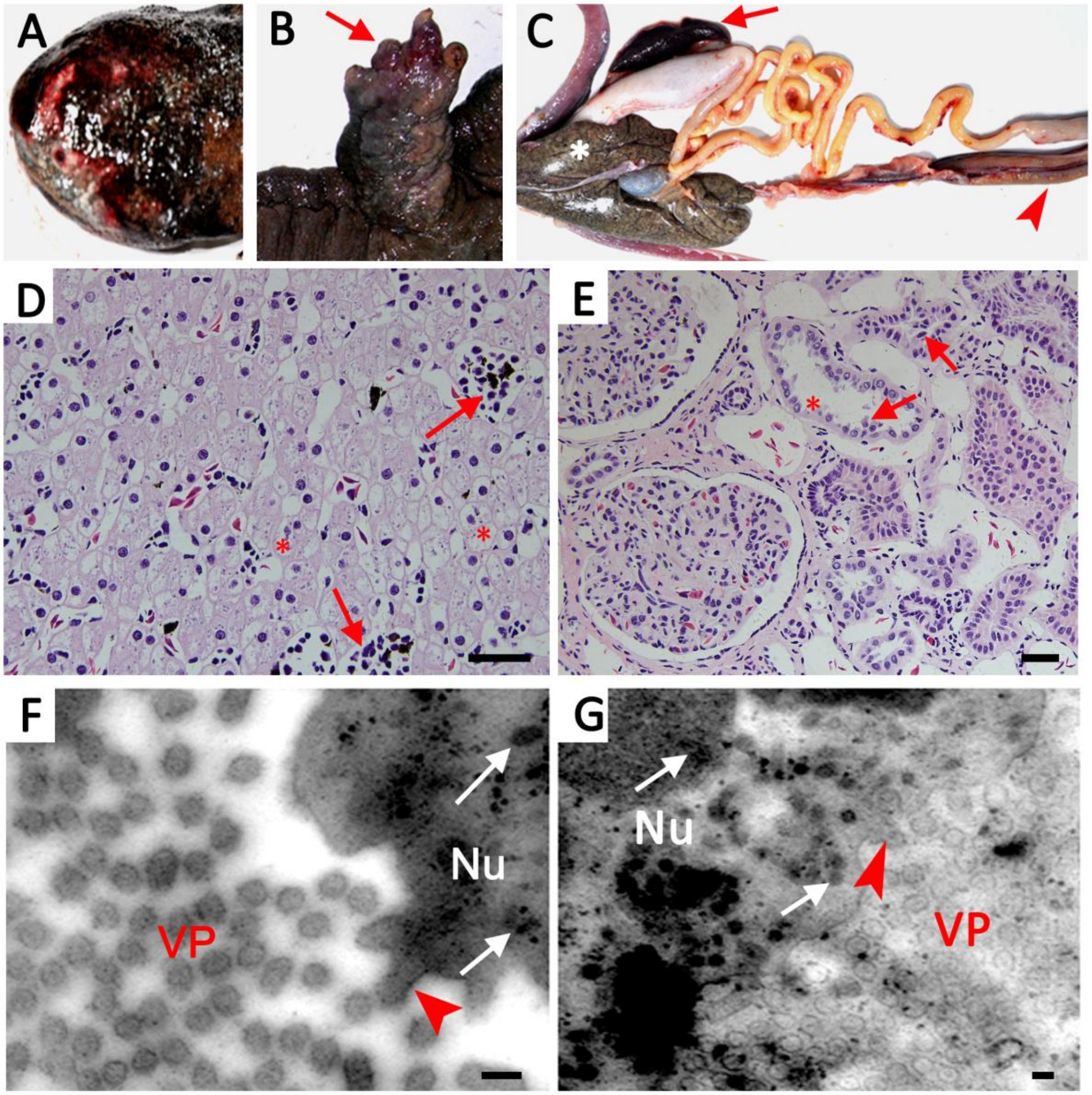

Figure 1. Gross anatomic and histologic changes in sick Chinese giant salamanders (*Andrias davidianus*), People's Republic of China, 2010. Viral particles are present in hepatocytes and renal cells. A) Palpebral hyperemia or edema; mouth pouch inflammation. B) Toe and tail necrosis (arrow). C) A sick Chinese giant salamander with a diseased spleen (arrow), a friable and gray-black liver (asterisk), and

mottled necrotic lesions in the kidney (arrowhead). D) Histologic changes in the liver of a sick Chinese giant salamander. Arrows indicate a large number of macrophages in enlarged liver sinusoids; asterisks indicate degenerating hepatocytes (hematoxylin and eosin stain; scale bar = 80 μm). E) Histologic changes in the kidney of a sick Chinese giant salamander. Asterisks indicate degenerating epithelial cells in renal tubules; arrows indicate epithelial cells in the renal tubule lumen (hematoxylin and eosin stain; scale bar = 80 μm). F) Electron microscopic image of viral particles in hepatocytes (scale bar = 200 nm). G) Electron microscopic image of viral viral particles in renal cells (scale bar = 200 nm). VP: viral particles in cytoplasm; Nu: nucleus; arrowheads, provirus in nuclear membrane; arrows, provirus in nucleus (scale bar = 200 nm).

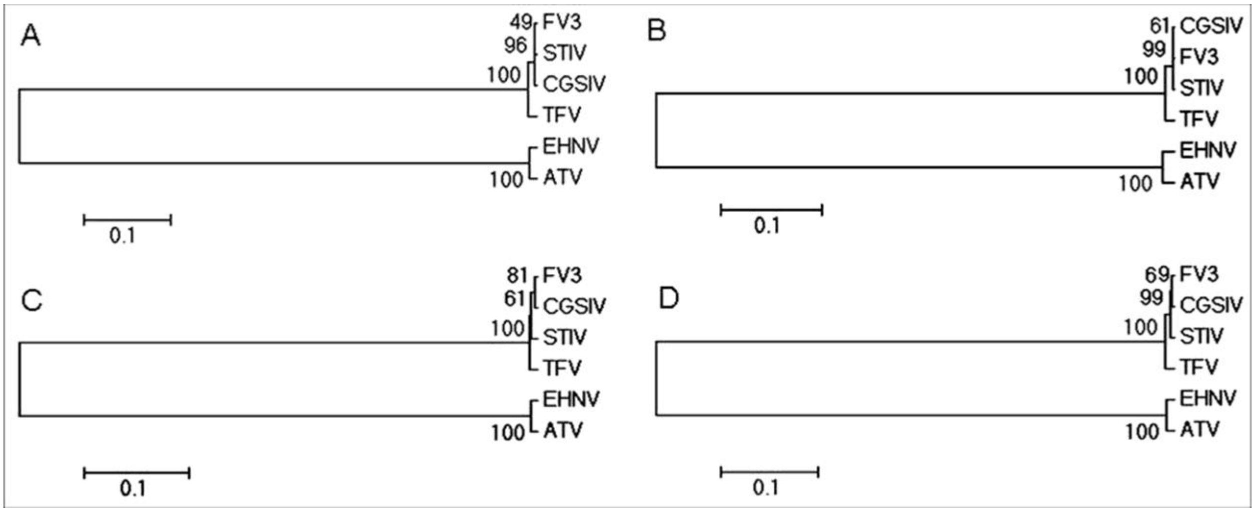

Figure 2. Neighbor-joining (NJ) tree comparison of gene sequences of viral isolates from Chinese giant salamander (CGSIV) and 5 known viral strains, People's Republic of China, 2010. Major capsid protein, ATPase, and thymidine kinase gene sequences of 5 known iridovirus stains were obtained from GenBank (frog virus 3 [FV3]: AY548484; soft-shelled turtle iridovirus [STIV]: EU627010; tiger frog virus [TFV]: AF389451; EHNV: FJ433873; Ambystoma tigrinum stebbensi virus [ATV]: AY150217) and corresponding sequences of the 15 CGSIV (GenBank accession nos. HQ829176, HQ829177, and HQ829178). All sequences were edited by using the DNASTAR 5.0 package (DNASTAR, Madison, WI, USA). These sequences were aligned with the ClustalX package (www.clustal.org) and truncated to equal bp, respectively. Insertions/deletions in the aligned sequences were excluded in the analyses. An NJ tree using the Kimura 2-parameter model with 1,000 bootstrap replicates was constructed to identify a possible phylogenetic tree in MEGA2.1 (www.megasoftware.net). A) NJ tree of major capsid protein sequences of CGSIV and the 5 viral strains. B) NJ tree of ATPase sequences of CGSIV and the 5 viral strains. C) NJ tree of thymidine kinase sequences of CGSIV and the 5 viral strains. D) NJ tree of major capsid protein + ATPase + thymidine kinase sequences of CGSIV and the 5 viral strains. The NJ tree showed that CGSIV was clustered in 1 lineage with FV3, STIV, and TFV, indicating that CGSIV belongs to the family *Iridoviridae*.
